# Reporting of key methodological issues in placebo-controlled trials of surgery needs improvement: a systematic review

**DOI:** 10.1016/j.jclinepi.2019.11.016

**Published:** 2020-03

**Authors:** Sian Cousins, Natalie S. Blencowe, Carmen Tsang, Ava Lorenc, Katy Chalmers, Andrew J. Carr, Marion K. Campbell, Jonathan A. Cook, David J. Beard, Jane M. Blazeby

**Affiliations:** a National Institute of Health Research (NIHR), Biomedical Research Centre at University Hospitals Bristol NHS Foundation Trust and the University of Bristol, Surgical Innovation theme and the Medical Research Council ConDuCT-II Hub for Trials Methodology Research, Bristol Centre for Surgical Research, Population Health Sciences, Bristol Medical School, Bristol, UK; b Division of Surgery, University Hospitals Bristol NHS Foundation Trust, Bristol, UK; c Nuffield Department of Orthopaedics, Rheumatology and Musculoskeletal Sciences, University of Oxford, Headington, Oxford, UK; d National Institute of Health Research (NIHR) Biomedical Research Centre, Oxford University Hospitals NHS Foundation Trust, Oxford, UK; e Royal College of Surgeons (England) Surgical Interventional Trials Unit (SITU), Botnar Research Centre, University of Oxford, Headington, Oxford, UK; f Health Services Research Unit, University of Aberdeen, Aberdeen, UK

**Keywords:** Surgery, Placebo surgery, Invasive procedures, Systematic review, Methodology

## Abstract

**Objectives:**

To examine key methodological considerations for using a placebo intervention in randomized controlled trials (RCTs) evaluating invasive procedures, including surgery.

**Study Design and Setting:**

RCTs comparing an invasive procedure with a placebo were included in this systematic review. Articles published from database inception to December 31, 2017, were retrieved from Ovid MEDLINE, Ovid EMBASE and CENTRAL electronic databases, by handsearching references and expert knowledge. Data on trial characteristics (clinical area, nature of invasive procedure, number of patients and centers) and key methodological (rationale for using placebos, minimization of risk, information provision, offering the treatment intervention to patients randomized to placebo, delivery of cointerventions, and intervention standardization and fidelity) were extracted and summarized descriptively.

**Results:**

One hundred thirteen articles reporting 96 RCTs were identified. Most were conducted in gastrointestinal surgery (*n* = 40, 42%) and evaluated minimally invasive procedures (*n* = 44, 46%). Over two-thirds randomized fewer than 100 patients (*n* = 65, 68%) and a third were single center (*n* = 31, 32%). A third (*n* = 33, 34%) did not report a rationale for using a placebo. Most common strategies to minimize patient risk were operator skill (*n* = 22, 23%) and independent data monitoring (*n* = 28, 29%). Provision of patient information regarding placebo use was infrequently reported (*n* = 11, 11%). Treatment interventions were offered to patients randomized to placebo in 43 trials (45%). Cointerventions were inconsistently reported, but 64 trials (67%) stated that anesthesia was matched between groups. Attempts to standardize interventions and monitor their delivery were reported in *n* = 7, (7%) and *n* = 4, (4%) trials, respectively.

**Conclusion:**

Most placebo-controlled trials in surgery evaluate minor surgical procedures and currently there is inconsistent reporting of key trial methods. There is a need for guidance to optimize the transparency of trial reporting in this area.

**What is new?**

**Key findings**
• Ninety-six placebo-controlled randomised trials of invasive procedures published up to December 2017 were identified. Most were conducted in gastrointestinal surgery (n = 40, 42%) and evaluated minimally invasive procedures (n = 44, 46%).
• There was limited and inconsistent reporting of information regarding the rationale for the study, information provision to patients, methods used to minimise risk and standardisation and monitoring of intervention delivery.

**What this study adds to what was known?**
• This review comprehensively identified all publications of placebo-controlled trials of invasive procedures and examined key methodological issues that have not been explored in previous reviews.

**What is the implication and what should change?**
• Findings highlight the need for reporting guidelines reflecting methodological best practice for the design and delivery of placebo-controlled studies of invasive procedures. This would include the minimum detail that researchers should state in trial protocols and in the reporting of results to ensure transparency and consistency across studies.

## 1 Introduction

Worldwide, there are at least 230 million invasive procedures performed annually [1]. Historically, the effectiveness of these procedures was rarely assessed through high-quality evidence from randomized controlled trials (RCTs). However, recent initiatives led by the Royal College of Surgeons of England [2], and investment by international funding bodies [[3], [4], [5]], has facilitated an increase in the number and quality of RCTs in surgery [6]. Despite these improvements, a remaining challenge relates to the design and delivery of placebo-controlled RCTs involving invasive procedures.

Placebo-controlled trials are considered the gold standard in the evaluation of health-care interventions because they optimize blinding of all stakeholders and thereby minimize bias. Placebo-controlled trials in surgery have been summarized in reviews focusing on treatment effectiveness [[7], [8], [9]], adverse events [7,8], challenges with recruitment and retention [10,11], the natural history of placebo responses [12,13], and the characteristics of the treatment interventions evaluated [9,10]. Collectively, these have established that they are feasible, although recruitment can be difficult, and that most trials evaluated less invasive minimal access treatments with few including a traditional “open”/“large cut” surgical procedures (i.e., most performed in endoscopic settings and without the use of general anesthesia).

Although these reviews are useful, several methodological features are unexplored. Reported rationales for the use of an invasive placebo intervention (rather than “standard care” or other treatment interventions) were not examined. This is important because of the potential risk associated with the delivery of invasive placebo interventions (and/or associated anesthesia). Furthermore, reviews did not examine how potential risks might be minimized and how patients are informed about placebo interventions, including whether enhanced consent processes and extra safeguards are used, as suggested by guidelines [[14], [15], [16], [17]]. The extent to which patients randomized to placebo groups are offered treatment interventions is also unknown and is important because it presents an additional degree of risk to patients and may influence recruitment. Finally, the reviews did not examine how to optimize placebo intervention design by consideration of the complexity of the treatment interventions and the additional diagnostic or therapeutic procedures delivered to patients as part of the treatment program (cointerventions) [18]. Specifically, the extent to which cointerventions are delivered in the placebo group and methods to standardize interventions and monitor fidelity to the protocol during the study were not studied.

The aim of this work was to conduct a systematic review to examine the reporting of these important methodological issues in placebo-controlled trials of invasive procedures.

## 2 Methods

A systematic review of RCTs comparing an invasive procedure with a placebo intervention was undertaken. Articles identified in a previous review [10] published between database inception and November 14, 2014, were included (*n* = 63). Searches using the same search terms [7] (Appendix 1) and electronic databases (Ovid MEDLINE, Ovid EMBASE, and CENTRAL [19]) were conducted to identify RCTs published from November 15, 2017, to December 31, 2017. Additional articles, with no restriction on publication date, were identified by hand searching references of included articles and expert knowledge.

### 2.1 Eligibility criteria

Articles reporting RCTs (including long-term follow-ups and protocols) comparing an invasive procedure with a placebo procedure in living humans were included. Pilot RCTs retrieved by the current search were included as a source of potentially useful information about the main trial methods. An invasive procedure was defined as any interventional procedure that changes the anatomy and requires a skin incision or the use of endoscopic techniques. The term “placebo” referred to a surgical placebo, a sham surgery, or a procedure intended to mimic the active intervention. RCTs that assessed medicinal products or dental interventions, nonrandomized studies, reviews, editorials, letters, and conference abstracts were excluded.

### 2.2 Screening articles

All articles retrieved from the current search (November 15, 2017–December 31, 2017) were imported into an EndNote database (EndNote™, version X8.0.2). Titles and abstracts were screened for eligibility, and full texts of potentially eligible articles were retrieved, and eligibility was confirmed. Screening was conducted independently by two reviewers (S.C. and K.C.).

### 2.3 Data extraction

Data were extracted from all articles, including study protocols and clinical trial registry reports, where available, by one reviewer using a standardized data extraction form. A second reviewer extracted data for 20% of articles to identify any potential systematic errors in data extraction; however, none were found. Where multiple articles related to the same trial included, they were grouped into a single set, and data extraction was then conducted on a “per trial” (rather than “per article”) basis.

Data were extracted on trial characteristics and key methodological areas of interest. Trial characteristics included year of publication, clinical area (e.g., gastrointestinal), number of study sites and patients randomized, and type of treatment intervention (e.g., endoscopic). Key methodological areas of interest included (i) rationale for use of an invasive placebo intervention; (ii) minimization of risk; (iii) information provision and informed consent; (iv) offering the treatment intervention to patients randomized to the placebo group; (v) delivery of cointerventions in the placebo group; and (vi) intervention standardization and fidelity.

#### 2.3.1 Rationale for use of an invasive placebo intervention

Reported rationales for using an invasive placebo intervention were extracted from the introduction section of articles. This included, but was not limited to, any report of its use to measure the placebo effect, reduce bias, or to determine the mechanism of action of the treatment intervention.

#### 2.3.2 Minimization of risk

Some information on processes to mitigate potential patient risk was extracted. This included details about technical skill (e.g., trial entry criteria such as having performed a minimum number of procedures), use of trial oversight or monitoring mechanisms (e.g., independent committees), or the use of explicit emergency unblinding protocols.

#### 2.3.3 Information provision and informed consent

Information provided to patients during informed consent regarding the use of a placebo intervention was extracted.

#### 2.3.4 Offering the treatment intervention to patients randomized to the placebo group

Whether the treatment intervention was offered to patients randomized to the placebo group at any point was extracted, including whether a rationale for this was reported.

#### 2.3.5 Delivery of co-interventions in the placebo group

It was reported whether the delivery of cointerventions (before, during, or after the invasive procedure) was matched between treatment and placebo groups. Specifically, whether anesthesia (not including premedications) was matched between groups, and if so, the type of anesthesia (local, general, or sedation) used was extracted.

#### 2.3.6 Intervention standardization and fidelity

Strategies to standardize treatment and placebo interventions and to monitor their delivery (e.g., videotaping) were captured.

## 3 Results

**Fig. 1:**
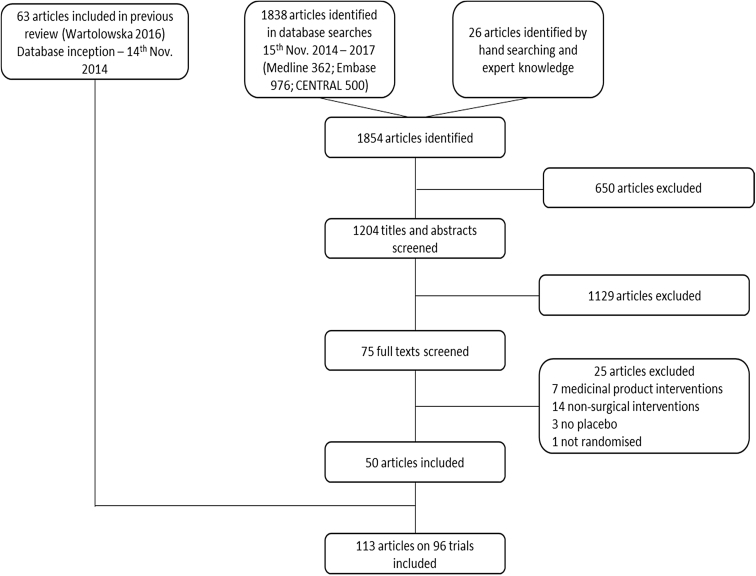
PRISMA diagram showing screening process of retrieved articles.

Current searches retrieved 1,854 articles, of which 75 full texts were screened and 50 were included (Fig. 1). Combined with 63 articles from the previous review [10], a total of 113 articles relating to 96 unique trials formed the final data set (Table 1). Most were conducted in gastrointestinal surgery (*n* = 40, 42%) and evaluated minimally invasive or endoscopic interventions (*n* = 44, 46%). Over two-thirds randomized fewer than 100 patients (*n* = 65, 68%) and approximately one-third involved a single center (*n* = 31, 32%).

**Table 1:** Characteristics of placebo-controlled trials of invasive procedures, *n* = 96

Characteristic	Number of RCTs (%)
Year of publication	
≤2000	29 (30)
2001–2010	39 (41)
2011–2017	28 (29)
Region	
Europe	40 (42)
United States	37 (36)
Australia	4 (4)
Canada	3 (3)
Asia	2 (3)
Not specified	1 (1)
Multiregion	9 (9)
Clinical area	
Gastrointestinal	40 (42)
Orthopedics & trauma	15 (16)
Oral and maxillofacial	10 (10)
Cardiothoracic	7 (7)
Ear, nose, and throat	6 (6)
Interventional cardiology	5 (5)
Neurosurgery	5 (5)
Other^a^	8 (8)
Number of centers	
1	31 (32)
2–5	17 (18)
6–10	7 (7)
>10	16 (17)
Not reported	25 (26)
Number of patients randomised^b^	
1–100	65 (68)
101–200	16 (17)
>200	14 (15)
Treatment intervention	
Endoscopic	44 (46)
Minimal access	21 (22)
Percutaneous	20 (21)
Open surgery	11 (11)

a Gynecology and obstetrics, *n* = 4; urology, *n* = 2; podiatry, *n* = 2.

b Not reported for 1 trial (recruitment not completed).

### 3.1 Rationale for the use of an invasive placebo intervention

Thirty-three trials (34%) did not report any rationale for using a placebo intervention. Approximately one-third (*n* = 27, 28%) justified the use of a placebo in terms of quantifying potential placebo effects. This included discussion of the limitations of previous non–placebo-controlled trials and known or expected placebo effects associated either with interventions of the same nature (invasive) or with the specific treatment intervention under evaluation [[20], [21], [22]]. The role of placebo interventions in reducing bias within RCTs was mentioned in nine trials (9%). These trials critiqued previous “open-label” studies evaluating the treatment intervention [20,[23], [24], [25], [26]] and highlighted the methodological advantages of using placebo interventions owing to their ability to control for bias [[27], [28], [29]]. Using a placebo intervention to help elucidate a treatment's mechanism of action was rarely explicitly reported as a rationale for use of a placebo. However, 10 trials (10%) implied this by stating an uncertainty over which treatment components were responsible for the mechanism of action.

### 3.2 Minimization of risk

Information about degree of operator skill was reported in 22 (23%) trials. Independent data monitoring was reported in 28 trials (29%). Of these, 17 explicitly reported monitoring of safety outcomes and 12 reported the use of formal interim analyses. Another trial [30] explicitly reported the use of a prospectively designed protocol under which patients would be unblinded to treatment received in the case of medical emergency.

### 3.3 Information provision and informed consent

Eleven trials (11%) reported details of information given to patients related to the use of a placebo intervention. Two required patients to write statements stating that they realized that they may receive placebo surgery and they further realized that this placebo surgery would not benefit their condition [31,32]. Similarly, one trial stated that patients were made to realize that they may receive only placebo surgery in which case their condition would be left untreated [33]. In contrast, one informed patients that the placebo procedure might improve symptoms [34]. Three trials [20,28,35] did not use the term “placebo” or “sham” in information given to patients, but rather they described the characteristics of both treatment and placebo procedures. Only two trials reported that patients were informed that they might not receive the treatment intervention [26,36], and one informed patients of the “blinded” nature of the study [37].

Only one trial [38] defined placebo surgery in information provided to patients (“placebo surgery simulates or mimics a surgical procedure, the person does not actually undergo the full surgical procedure.”) This placebo-controlled feasibility trial highlighted as an implication for practice that “patient information leaflets within placebo-controlled trials should explicitly state that while benefit might be seen within a placebo group, the underlying mechanism of the placebo has no known direct effect” [39].

### 3.4 Offering the treatment intervention to patients randomized to the placebo group

Patients allocated to the placebo group were offered the treatment intervention in 43 (45%) trials. Seven (7%) were crossover trials where all patients received both treatment and placebo interventions. This included one trial with a four-group design in which the order that interventions were delivered was randomized [40]. At least one rationale for offering treatment to the placebo group was given by 16 (37%). Seven stated that the treatment intervention was offered if patients had continuing symptoms. In five, ethical reasons were specified, for example, “for ethical reasons, patients in the sham group had callosities debrided before leaving the clinic” [41]. Some trials also reported that offering the treatment to all patients was a strategy to optimize recruitment and retention (*n* = 5), with one stating that “asking patients to remain untreated risked their noncompliance with follow-up evaluation” [42]. Methodological advantages of this approach were cited by three trials; patients could act as their own controls in a trial with a crossover design [43]; delivery of treatment after placebo “provided the opportunity to investigate whether [the treatment intervention] would be of secondary benefit after successful or failed primary (sham) treatment” [40]; and offering patients the treatment intervention may reduce the likelihood of patients seeking treatments outside of the study protocol [42].

### 3.5 Delivery of co-interventions in the placebo group

Details about any co-intervention delivered preprocedure, periprocedure, and postprocedure were reported in 45 (47%), 31 (32%), and 64 (67%) of trials, respectively. Explicit matching of co-interventions between treatment and placebo groups was reported in 42/45 (preprocedure), 27/31 (peri-procedure) and 61/64 (postprocedure). Anesthesia was matched between groups in 64 trials (67%). Types of anesthesia used in these trials included sedation (*n* = 23, 36%), local (including regional blocks) (*n* = 20, 31%), general (*n* = 13, 20%), and combinations (*n* = 7, 11%). In one study, it was unclear which type of anesthesia was used.

### 3.6 Intervention standardization and fidelity

Attempts to standardize trial interventions were reported in 6 trials (6%). Four trials reported that interventions were delivered in accordance with a protocol [23,[44], [45], [46]]: One reported the use of a standardized protocol for delivering treatment interventions only [46]; one specified both treatment and placebo interventions were delivered using the same standardized protocol [44]; one specified that all operators adhered to a standardized protocol but gave no further detail [23]; and one specified that surgeons were allowed to apply their own techniques within the limits of the protocol [45]. Another trial reported that consensus on a standard intervention approach was reached by all participating investigators and a treatment consultant at a prestudy meeting [47], and one reported that uniformity of intervention delivery was ensured by the use of just one clinician to deliver all interventions [48].

Four trials (4%) reported strategies to monitor the delivery of interventions (fidelity). In three, the interventions were videotaped [33,45,49] and the other stated that “adherence to technique was monitored by the Study Chairman” [47], although no further details were given.

## 4 Discussion

Placebo-controlled trials of invasive procedures are controversial and can be challenging to conduct. This systematic review identified 96 such trials. Most evaluated minimally invasive endoscopic procedures, with only 11 trials of traditional “open” surgeries involving an incision. Owing to the potential for patient risk in these trials, careful consideration is needed to justify their use [14,16,17]. Despite this, a third of trials did not provide a rationale for the placebo-controlled design and strategies to minimize potential risk to patients were poorly reported. Contradictory information about the use of an invasive placebo intervention was also given to patients across trials; some trials informed patients the placebo intervention would not benefit their condition [[31], [32], [33]], whereas another specified that it would [34]. A large proportion of trials also offered the treatment intervention to patients randomized to the placebo group. Although ethical reasons were sometimes cited for this, further consideration of the potential added risk of offering patients an additional treatment with unproven benefit is required. There is therefore a need for better guidance for the design, conduct, and reporting of placebo RCTs in invasive procedures, including surgery.

In agreement with previous reviews [9,10], most RCTs with an invasive placebo intervention evaluated minimally invasive treatments. Although the RCTs evaluated treatments from across specialties, most were conducted in gastro-intestinal surgery, where minimally invasive techniques are commonly used (e.g., endoscopic procedures). In addition, in keeping with previous reviews of surgical RCTs [[50], [51], [52]], the standard of reporting in the RCTs across the examined methodological areas of interest was inconsistent. One area that was poorly reported was the rationale for using an invasive placebo comparator within the RCT. Guidance issued by the American Medical Association [14], specifies the ethical use of invasive placebo interventions only in cases where no other trial design will yield the requisite data. However, one-third of trials did not report any rationale for the use of an invasive placebo comparator, in place of standard or no treatment comparators. This guidance and others [[15], [16], [17]] also highlight the need for rigorous informed consent processes in placebo surgical trials, including providing patients with information that describes the differences between treatment and placebo interventions and the risk posed by each. Despite this, the reporting of information provision to patients regarding the use of an invasive placebo intervention was poor. In addition, although it is a key issue to consider in RCTs with an invasive placebo comparator, strategies to minimize potential risk to patients, such as additional oversight mechanisms, were poorly reported. However, it is possible that clinical methods to minimize risk (e.g., highly selecting patients or consideration of different modes of anesthesia) were used in the trials.

Findings highlight the need for reporting guidelines reflecting methodological best practice for the design and delivery of placebo-controlled studies of invasive procedures. As with existing reporting guidelines for RCTs [53], this would include the minimum detail that researchers should state in trial protocols and in the reporting of results to ensure transparency and consistency across studies. Key methodological areas that currently have considerable gaps in the quantity and quality of reported information include justification for use of an invasive placebo, minimization of patient risk, patient information provision regarding placebo use, rationales for offering patients to the placebo group the treatment intervention, and standardization of interventions across patients/trial sites.

The current review comprehensively identified all publications of placebo-controlled trials of invasive procedures using systematic methods, including handsearching references and expert knowledge. However, additional details about the RCTs may possibly have been gained from contacting trial teams directly, and this may have provided further insight into the design and delivery of these trials. Although this was not practically possible for the current review, it is important that future work to improve the design and reporting of these RCTs involves key stakeholders, including trialists, clinicians, and journal representatives. We also acknowledge that assessments of risk of bias [54] may have provided valuable information about the quality of the included RCTs [55]. However, given the focus of this review on specific key methodological issues not reported in previous reviews, it was not included. Furthermore, it should be highlighted that the inconsistent reporting of these trials may in part reflect journal restrictions on manuscript length, a limitation common to all reviews of published articles.

This review demonstrates that placebo-controlled trials of invasive procedures are being undertaken in a range of surgical specialties (but especially in gastro-intestinal surgery); however, they are poorly reported, an issue common to all RCTs in surgery [[50], [51], [52]]. In addition to the development of reporting guidelines, more work is needed to develop guidance to optimize the design and conduct of RCTs in this field, and it is important that this be carried out in consultation with patients and key stakeholders to ensure acceptability. Placebo-controlled trials have the potential to answer important clinical questions [20,31]; however, there is a need to ensure standardization and implementation of appropriate methods across RCTs.

## CRediT authorship contribution statement

**Sian Cousins:** Conceptualization, Methodology, Validation, Formal analysis, Investigation, Data curation, Writing - original draft, Writing - review & editing, Supervision, Project administration. **Natalie S. Blencowe:** Conceptualization, Methodology, Validation, Formal analysis, Investigation, Data curation, Writing - original draft, Writing - review & editing, Supervision, Project administration, Funding acquisition. **Carmen Tsang:** Validation, Formal analysis, Investigation, Writing - original draft, Writing - review & editing. **Ava Lorenc:** Formal analysis, Investigation, Writing - review & editing. **Katy Chalmers:** Methodology, Validation, Formal analysis, Investigation, Data curation, Writing - review & editing. **Andrew J. Carr:** Conceptualization, Methodology, Writing - review & editing, Supervision. **Marion K. Campbell:** Conceptualization, Methodology, Writing - review & editing, Supervision. **Jonathan A. Cook:** Conceptualization, Methodology, Writing - review & editing, Supervision. **David J. Beard:** Conceptualization, Methodology, Writing - review & editing, Supervision. **Jane M. Blazeby:** Conceptualization, Methodology, Validation, Investigation, Data curation, Writing - original draft, Writing - review & editing, Supervision, Project administration, Funding acquisition.
