# Supplementary material for: Reporting of key methodological issues in placebo-controlled trials of surgery needs improvement: a systematic review
Source: J Clin Epidemiol. 2020 Mar;119:109–16. doi: 10.1016/j.jclinepi.2019.11.016 (PMC7066579; doi:10.1016/j.jclinepi.2019.11.016)
Supplement: Appendix A [file mmc1.docx]

**Appendix A – Search terms uses in Ovid MEDLINE, Ovid EMBASE and CENTRAL databases**

**Ovid MEDLINE**

1. Clinical trial/

2. Randomized controlled trial/

3. Randomization/

4. Rct.tw.

5. random allocation.tw.

6. Randomly allocated.tw.

7. Allocated randomly.tw.

8. Randomized Controlled Trials as Topic/

9. randomized controlled trial/

10. Double Blind Method/

11. Single Blind Method/

12. clinical trial/

13. controlled clinical trial.pt.

14. randomized controlled trial.pt.

15. clinical trial.pt.

16. exp Clinical Trials as topic/

17. or/1-16

18. PLACEBOS/

19. placebo$.tw.

20. sham.tw.

21. immitation.tw.

22. placebo effect$.tw.

23. or/18-22

24. surgery.tw.

25. surgical.tw.

26. arthroscopy.tw.

27. endoscopy.tw.

28. transplantation.tw.

29. $scopy.tw.

30. $scopic.tw.

31. laparoscopy.tw.

32. Meta-Analysis as Topic/

33. meta analy$.tw.

34. metaanaly$.tw.

35. Review/

36. Comment/

37. Letter/

38. Editorial/

39. animal/

40. dose$.tw.

41. pre$medication.tw.

42. an$esthesia.tw.

43. an$esthetic$.tw.

44. antibiotic$.tw.

45. steroid$.tw.

46. prophylaxis.tw.

47. prevention.tw.

48. preoperative.tw.

49. preanaesthetic$.tw.

50. pre$emptive.tw.

51. pre-operative.tw.

52. post-operative.tw.

53. postoperative.tw.

54. post$surgery.tw.

55. (analgesic adj trial).tw.

56. oral$.tw.

57. acupuncture.tw.

58. acupressure.tw.

59. scar.tw.

60. infection.tw.

61. dental.tw.

62. post$surgical.tw.

63. pre$surgical.tw.

64. case report.tw.

65. case study.tw.

66. pacing.tw.

67. stimulation.tw.

68. growth factor$.tw.

69. hormon$.tw.

70. or/24-31

71. or/32-69

72. 17 and 23

73. 72 and 70

74. 73 not 71

Ovid EMBASE

1. Clinical trial/

2. Randomized controlled trial/

3. Randomization/

4. Single blind procedure/

5. Double blind procedure/

6. Crossover procedure/

7. Randomi?ed controlled trial$.tw.

8. Rct.tw.

9. random allocation.tw.

10. Randomly allocated.tw.

11. Allocated randomly.tw.

12. (allocated adj2 random).tw.

13. Single blind$.tw.

14. Single blind$.tw.

15. or/1-14

16. Placebo$.tw.

17. placebo effect$.tw.

18. sham.tw.

19. placebo.tw.

20. or/16-19

21. surgery.tw.

22. surgical.tw.

23. arthroscopy.tw.

24. endoscopy.tw.

25. $scopy.tw.

26. $scopic.tw.

27. laparoscopy.tw.

28. transplantation.tw.

29. or/21-28

30. letter/

31. Review/

32. animal/

33. editorial/

34. ((meta adj analy$) or metaanalys$).tw.

35. (analgesic adj trial).tw.

36. meta$analysis.tw.

37. dose$.tw.

38. oral$.tw.

39. orally.tw.

40. dental.tw.

41. pre$medication.tw.

42. pre$surgical.tw.

43. post$surgical.tw.

44. pre$surgery.tw.

45. post$surgery.tw.

46. antibiotic$.tw.

47. an$esthetic$.tw.

48. steroid$.tw.

49. peri$operative.tw.

50. pre$emptive.tw.

51. pre$an$esthetic$.tw.

52. post$operative.tw.

53. prophylaxis.tw.

54. prevention.tw.

55. acupuncture.tw.

56. accupressure.tw.

57. scar$.tw.

58. infection$.tw.

59. acupressure.tw.

60. pre$operative.tw.

61. growth factor$.tw.

62. pacing.tw.

63. stimulation.tw.

64. hormon$.tw.

65. case report$.tw.

66. case study.tw.

67. or/30-66

68. 15 and 20

69. 68 and 29

70. 69 not 67

**Cochrane Central Register of Controlled Trials**

http://onlinelibrary.wiley.com/o/cochrane/cochrane_clcentral_articles_fs.html

(placebo OR placebo effect OR sham OR imitation):ti,ab,kw and (surgery OR surgical OR laparoscopy OR endoscopy

OR arthroscopy OR transplantation OR scopy):ti,ab,kw and (clinical trial OR randomised clinical trail OR RCT OR

randomised controlled trial OR randomisation ):ti,ab,kw not (drug OR dental OR oral OR infection OR steroids OR

hormones OR growth factor OR prophylaxis OR anaesthesia OR pre-surgical OR post-surgical OR pre-emptive OR

post-operative OR preoperative OR antibiotics OR acupuncture OR acupressure OR scar OR infection OR

prevention):ti,ab,kw not (review OR animal OR stimulation):ti,ab,kw in Trials
